# Novel highly efficient ternary ZnO wrapped PPy-NTs/g-C_3_N_4_ nanocomposite as an epoxy coating for corrosion protection

**DOI:** 10.1038/s41598-023-48557-9

**Published:** 2023-12-04

**Authors:** Heba A. El-Sabban, M. A. Deyab

**Affiliations:** 1 Egyptian Petroleum Research Institute (EPRI), Nasr City, Cairo Egypt; 2 Central Analytical Laboratories, Egyptian Petroleum Research Institute (EPRI), 1 Ahmed El Zomor St., Nasr City, 11727 Cairo Egypt

**Keywords:** Chemistry, Materials chemistry, Physical chemistry

## Abstract

The main goal of this study is to develop an epoxy coating coupled with an organic–inorganic hybrid nanocomposite that can be used as a corrosion-inhibiting pigment on carbon steel. Herein, polypyrrole nanotubes (PPy-NTs), polypyrrole nanotubes/g-C_3_N_4_ (PPy-NTs/g-C_3_N_4_) and novel nano-composite polypyrrole nanotubes/g-C_3_N_4_/ZnO (PGZ) were prepared by facile wet impregnation approach. The developed pigments were investigated using XRD, FTIR, FE-SEM equipped EDS. Electrochemical impedance spectroscopy (EIS) and polarization measurements were used to assess the behavior of the prepared pigments on the anticorrosion performance of epoxy resin coatings. EIS experiments revealed that introducing nano-pigments to neat coatings enhanced the epoxy resin and charge transfer resistance. The anticorrosion performance of the three nano-pigments was assessed as follows: PGZ ˃ PPy-NTs/g-C_3_N_4_˃ PPy-NTs.

## Introduction

Metal corrosion represents an emergent problem when metal components undergo damage by chemical or electrochemical interactions. In the past few decades, organic coating developed as a highly effective approach for metal protection. Epoxy coating is one of metal anticorrosion technology's most established and operative methods. The benefits of waterborne epoxy coatings for industries include simplicity of application, lowering flammability and health risks when dealing with paint, less odor, simple washing with water in place of organic solvents, Excellent chemical inertness and good hydroxyl group adhesion to metallic surfaces^1–4^. However, these coatings are hydrophilic, and corrosive ions could easily permeate through them. Additionally, the pores and flaws produced by the aqueous epoxy coatings' curing process may help corrosive chemicals diffuse into the coating. Because of this, even though aqueous epoxy coatings have been commercially available for more than 40 years, their usage is limited in corrosive environments, especially in marine environments^5–8^.

Nanotechnology is a promising approach that can efficiently overcome these drawbacks, enhance barrier effectiveness of the aqueous epoxy coatings, and produce nanocomposite coatings with long-term corrosion resistance^9–11^. For instance, polymeric nano-reinforced coatings have drawn much scientific attention as a practical way to protect metal surfaces from corrosion and fouling, particularly steel. Corrosion protection for bulky materials can be improved depending on the mechanical, physical, chemical features of materials in the nano-range^12^. It will be possible to attain this good barrier performance by lowering porosity, zigzagging the diffusion pathway, and making nanoparticles soluble in the polymer matrix^13^.

Among the most often utilized inorganic nanoparticles, nano-zinc oxide serves various functions and is used to create multifunctional nano coatings^14–18^. It has great dispersion with no aggregations, high hardness, a low refractive index, and hydrophobic enhancement^2,19–21^. On the other hand, intrinsically conducting polymers (ICPs) have received much attention due to their numerous potential uses in water treatment, sensors, supercapacitor electrodes, biological industries, and corrosion prevention^22–26^. ICPs can provide a barrier layer of protection and release coating inhibitors in the anticorrosion sector^12^. Additionally, they can undergo redox processes and thereby allow for the binding and ejecting of counter-ions (dopants) in response to the variation of the metal surface potential stimulated by local electrochemical reactions due to the corrosion^27^.

Polypyrrole (PPy) is the most promising polymer among the ICPs because of its simple polymerization, mechanical durability, improved biocompatibility, and tunable electrical properties^13^. The anticorrosion capabilities of the coatings are enhanced by selecting the right synthesis parameters^28–31^.

Two-dimensional with sheet-like nanomaterials with, high specific surface areas, chemical constancy, and great mechanical strength, such as graphitic carbon nitride (g-C_3_N_4_) nanosheets, have been incorporated inside the polymer coatings as nanofillers to decrease the defects, increase barrier resistance, and enhance the mechanical performance of the coatings^32–34^. Compared to graphene, the g-C_3_N_4_ is significantly more stable physic-chemically and contains a lot of nitrogen in the plane of the molecule. By possessing good mechanical stability and chemical resistance features, the reinforcement of g-C_3_N_4_ to a base epoxy matrix could greatly improve the mechanical strength of the final functionalized coating. In the past two years, some of the researchers are focused on their research to utilize the novel g-C_3_N_4_ as reinforcement materials for improving the overall performance of the polymer matrix composite materials^35–38^.

Based on the abovementioned studies, a straightforward wet impregnation approach was applied to prepare highly efficient ZnO/PPy-NTs/g-C_3_N_4_ nanocomposite as a novel epoxy coating. The ZnO/PPy-NTs/g-C_3_N_4_ nanocomposites were thoroughly characterized, and their uniform distribution and structure were confirmed. Finally, the produced composite was further reinforced with epoxy coatings, and the corrosion-resistant performance of the developed nanocomposite/epoxy coating was methodically evaluated.

## Experimental

### Preparation of ZnO nanoparticles (ZnO NPs)

Zinc oxide nanoparticles are prepared by the sol–gel method. 0.5 g of Zinc acetate dihydrate was typically dissolved in 50 mL ultrapure water. Then, the NH_4_OH solution dropped until pH 8 was reached, and the mixture kept stirring for 3 h until a white sol was formed. After aging the white sol for 48 h, the gelatinous phase was produced. Afterward, the gelatinous substance was dried at 100 °C. Finally, the dried powder was calcined at 500 °C for 2 h forming ZnO nanoparticles.

### Preparation of polypyrrole nanotubes (PPy-NTs)

PPy-NTs were synthesized via the chemical oxidation polymerization technique by sodium bis(2-ethylhexyl) sulfosuccinate emulsions (AOT) reverse (water-in-oil) emulsions with minor modifications^39^. Typically 0.7 wt% of PPy: FeCl_3_ was added to the AOT reversed micelle phase and stirred for 1 h. Then the formed product was rinsed against ethanol and kept under vacuum drying. AOT reverse cylindrical micelles were employed as the soft template. When the product was washed thoroughly with excess ethanol, AOT and other residual reagents were removed, leaving the PPy-NTs.

### Preparation of g-C_3_N_4_ nanosheets

The bulk g-C_3_N_4_ was prepared using thiourea as a starting material. 4 g of thiourea powder was typically thermally treated at 500 °C under Ar gas flow^40^. For the preparation of g-C_3_N_4_ nanosheets, typically, 1 M HNO_3_ and 2 g of bulk g-C_3_N_4_ were mixed and agitated for 24 h at 90 °C. After treatment, a uniformly mixed suspension was centrifuged to remove the supernatant. The residue was then rinsed twice with ultrapure water before being vacuum-dried for 24 h at 70 °C. It's significant to note that during the acidic treatment process, g-C_3_N_4_ sheets were oxidized and exfoliated by the entrance of HNO_3_ particles between the interlayers, resulting in the oxidation of the C-N bonds of the triazine units and the introduction of oxygen-functional groups. The protonated g-C_3_N_4_ sheets were then further exposed to exfoliation by ultra-sonication in ultrapure water for 30 min. The resultant dispersion was subsequently centrifuged and thoroughly washed with ultrapure water and kept drying at70 °C.

### Preparation of ternary ZnO/PPy-NTs/g-C_3_N_4_ hybrid nanocomposite (ZPG)

**Figure 1:**
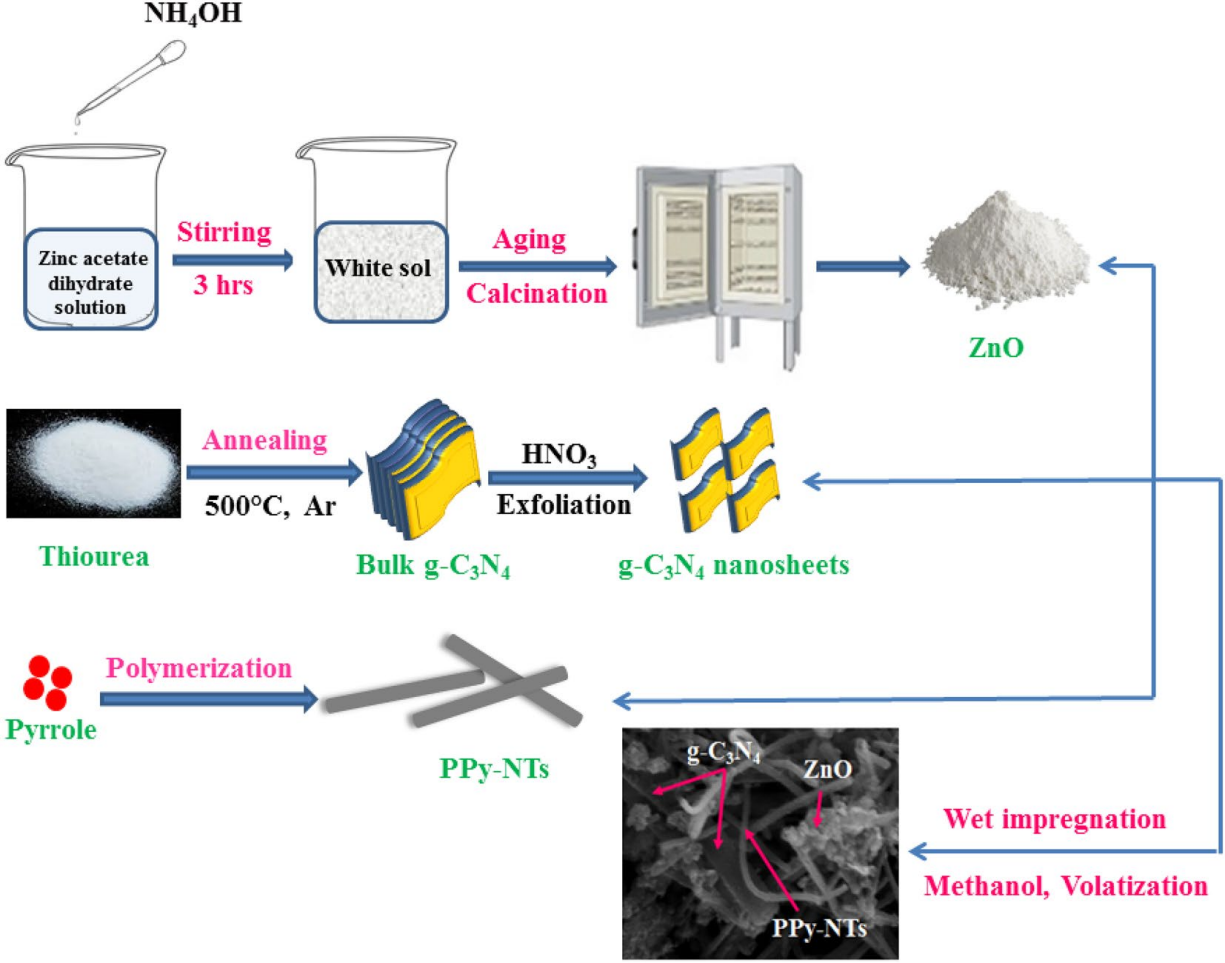
Schematic illustration of ternary ZnO/PPy-NTs/g-C_3_N_4_ fabrication procedure.

The straightforward wet impregnation approach was used to prepare ZnO/PPy-NTs/g-C_3_N_4_ nanocomposite (ZPG). Typically, 1:1:1 wt. % of ZnO:PPy-NTs:g-C_3_N_4_ are mixed with 50 mL methanol and then subjected to sonication for 2 h. After that, the suspension was stirred until the volatilization of methanol, and the product was kept dried at 60 °C. Finally, the schematic representation of the ternary ZnO/PPy-NTs/g-C_3_N_4_ hybrid nanocomposite (ZPG**)** fabrication procedure is displayed in Fig. 1.

### Characterization of the as-prepared materials

Field emission scanning electron microscopy (FE-SEM-Quanta-250 model) was used for morphology investigation. The chemical composition of the prepared samples was examined by X-ray diffraction (XRD-X’Pert Pro PANalytical model). Infrared spectroscopy investigates the function groups (IR-Perkin Elmer model). Examining oxidation states was done using an X-ray photoelectron (XPS-Thermo Fisher, Scientific model).

### Preparation of the nanocomposite coatings

Acetone (a solvent) and hardener (resin/hardener with volume ratio = 2) are used to disperse 1.0% of as-prepared materials inside epoxy resin (Bisphenol epoxy resin- PC™ Resin 80–80% solid content) to create nanocomposite coatings. To achieve the required nanoparticle size, this mixture was stirred mechanically (speed = 1500 rpm) for 2.0 h, sonicated for 3 h, and then ground in a YLK Mini planetary ball mill for 3 h. Vacuum evaporation was used to remove the acetone. The clean substrate was coated with nanocomposite coatings using a film applicator. The coating micrometre (Mitutoyo.co) was used to measure the dry film thickness, which was 53 ± 2.3 μm. The hybrid composite concentration calculated during coating preparation based on the weight of the solid components present in the coating formulation. The volatile components, such as solvents or reactive diluents, are not considered in the calculation. The concentration is expressed as a percentage of the total weight of the dry components**.**

### Electrochemical measurements

Electrochemical impedance spectroscopy (EIS) and polarization measurements were used to assess the anticorrosion characteristics of coatings made of nanocomposite materials. Three electrode cells and Gamry 3000 potentiostat/galvanostat were used to conduct the experiments. The electrode cell arrangement included a saturated calomel electrode (SCE, reference electrode), coated carbon steel (working electrode), and platinum (auxiliary electrode–surface area = 4.6 cm^2^). The composition of carbon steel is (wt. %): C(0.24%), P(0.028%), Mn(1.3%), Cr(0.26%), Cu (0.47%), Ni (0.28%), S(0.027%); Fe(balance%). The surface area of working electrode is 2.8 cm^2^. Before applying coatings, the carbon steel samples were abraded with SiC sheet (grads = 500, 800, and 1200) and washed with acetone and a solution of distilled water. The EIS measurements were conducted at open circuit potential (OCP) with voltage amplitudes of 10 mV in the frequency range of 0.01 Hz to 100 kHz. For EIS data fitting, the EC-Lab program was utilized. Polarization curves were recorded in a voltage range of ± 250 mV vs. OCP at a constant sweep rate of 1.0 mV s^−1^. The electrolyte is 3.5% NaCl solutions (pH = 7.8, conductivity = 52 mS cm^−1^).

## Results and discussions

### Structural and morphological characteristics

**Figure 2:**
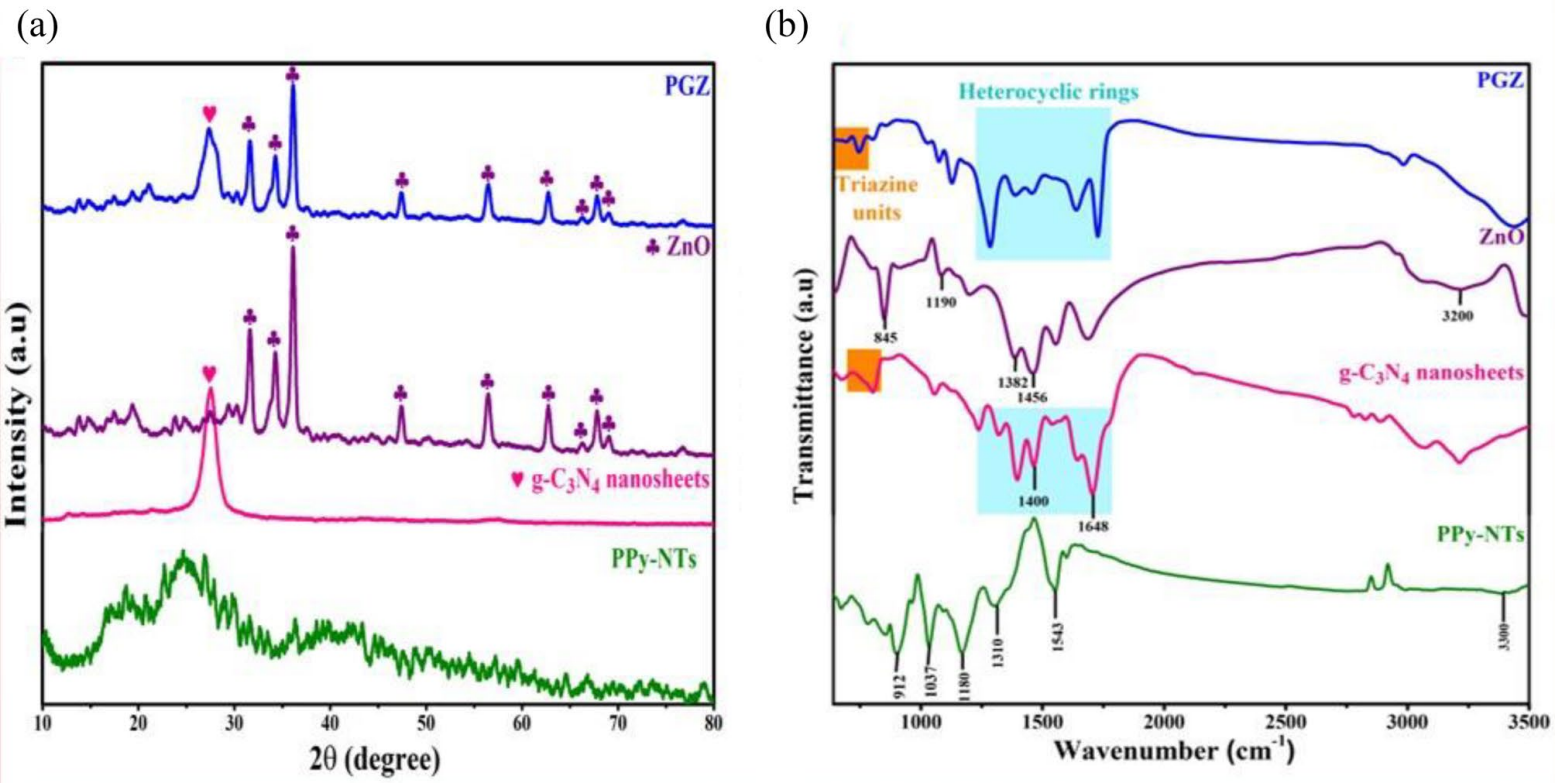
(**a**) XRD pattern, and (**b**) FTIR spectra of PPy-NTS, bulk g-C_3_N_4_, g-C_3_N_4_ nanosheets, ZnO, ZPG hybrid nanocomposite.

The XRD patterns and FTIR spectra of ZnO, PPy-NTs, bulk g-C_3_N_4_, g-C_3_N_4_ nanosheets, and ternary ZPG nanocomposite are presented in Fig. 2a, b.

Regarding the XRD pattern (Fig. 2a), PPy-NTs displayed an amorphous nature with a non-crystalline (wide) peak seen at roughly 2θ of 25°, displaying the distinctive properties of the matrix (Fig. 2a)^41–43^. For bulk g-C_3_N_4_ (Fig. S1), two distinctive diffraction peaks were identified at 27.3° and 13.3°, respectively^40,44^ These peaks are attributed to the (002) crystal faces of interlayer aromatic graphitic-like structures. Notably, the peak at 27.3° for g-C_3_N_4_ nanosheets (Fig. 2a) was slightly displaced to 27.6° compared to that of bulk g-C_3_N_4_ sheets, indicating a smaller interlayer spacing among the individual sheets that were initially formed^45,46^. Additionally, due to the sharp reduction in the g-C_3_N_4_ layers' planar distance during protonation and exfoliation processes, the strength of the 13.3° peak for the nanosheets is less obvious and significantly reduced compared to that of bulk g-C_3_N_4_^45^, displaying a single-layered structure. Concerning zinc oxide (Fig. 2a), several characteristic peaks were detected at 31.7°, 34.3°, 36.2°, 47.4°, 56.5°, 62.7°, 66.3°, 67.8°, 69°, and 76.8°, corresponding to the (100), (002), (101), (102), (110), (103), (200), (112), (201), and (202) miller indices that are assigned to hexagonal ZnO (*a* = 3.25, *b* = 3.25, and *c* = 5.21 Å), as indexed by (ICDD: 01-076-0704) standard data^47^. The main characteristic diffraction peaks of g-C_3_N_4_ nanosheets and ZnO are observed for the ternary ZPG nanocomposite (Fig. 2a). Remarkably, the intensity of g-C_3_N_4_ nanosheets crystal faces and ZnO was reduced in the prepared ZPG due to the amorphous nature of PPy-NTS_,_ which suggests the fruitful development of the hybrid nanocomposite by implanting the PPy-NTs inside the prepared matrix^48^.

Figure 2b depicts the FTIR spectra of the prepared samples. Concerning PPy-NTs, the bands at 1543 cm^−1^ is attributed to C=C stretching mode of the pyrrole ring, respectively^49^. N–H stretching vibration has been credited for the broad band at 3300 cm^−1^, whereas the bands at 1310 cm^−1^ and 1180 cm^−1^, respectively, were accredited to anti-symmetrical C–N and C–H stretching^50,51^. The Peaks at 1037 cm^−1^ and 912 cm^−1^ are connected to PPy’s doped state^50^. The g-C_3_N_4_ exhibits a number of strong distinctive peaks attributed to the stretching vibrational modes of the heterocyclic rings between 1200 and 1700 cm^−1^, as well as a peak at 808 cm^−1^ attributed to the stretching vibrational modes of triazine units^40^. The C_3_N_3_ stretching vibrational mode is attributed to the peak located at 1400 cm^−1^. The O–H bending and stretching vibrational modes is also responsible for the peaks located at 1648 cm^−1^^52^. According to ZnO, the typical characteristic peaks can be detected at, 845, 1190, 1382, and 1456 cm^−1^^53^. The broad peak at about 3200 cm^−1^ is assigned to O–H stretching vibration of water^54^. It is clear from that FTIR spectrum of the PGZ composite represented the mutual influence of PPy-NTs, g-C_3_N_4_, and ZnO by comparing with the corresponding distinctive peaks of PPy-NTs, g-C_3_N_4_, and ZnO. The peak strength and position, however, showed spectroscopic peak deviations, indicating interaction rather than a simple blending of the counterparts. Results from FT-IR confirm the development of PGZ nanocomposite that agrees well with results of the X-ray diffraction pattern.

**Figure 3:**
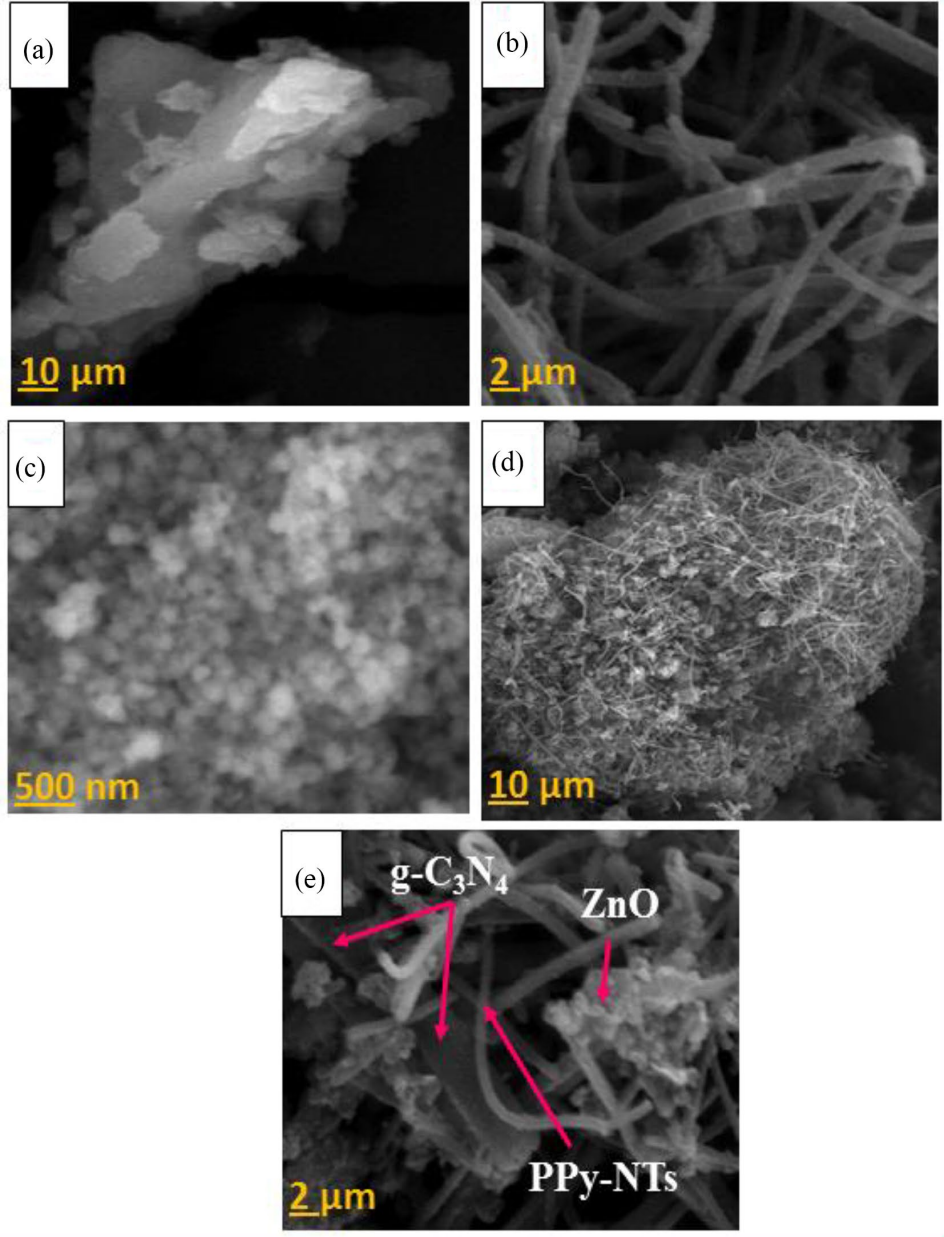
FE-SEM images of (**a**) g-C_3_N_4_ nanosheets, (**b**) PPy-NTs, (**c**) ZnO NPs, and (**d**, **e**) ZPG hybrid nanocomposite at different magnifications.

The morphological characteristics of the as-prepared g-C_3_N_4_ nanosheets, PPy-NTs, ZnO NPs, and ZPG hybrid nanocomposite were investigated using FE-SEM. Figure 3a displays the SEM image of g-C_3_N_4_, revealing highly exfoliated graphene-like sheets. PPy-NTs exhibit a 1D uniform hollow nanotube-like structure (Fig. 3b). It is noteworthy that numerous nanotubes are wrapped around one another due to—interactions with the backbone chains of the PPy-NTs. From Fig. 3c, it can also be seen that the morphology of the ZnO nanoparticles displays -a spherical shape. Regarding ZPG nanocomposite at two different magnifications (Fig. 3d, e), intimate interaction between g-C_3_N_4_ nanosheets, PPy-NTs, and ZnO NPs can be detected, demonstrating the successful construction of hybrid nanocomposite.

**Figure 4:**
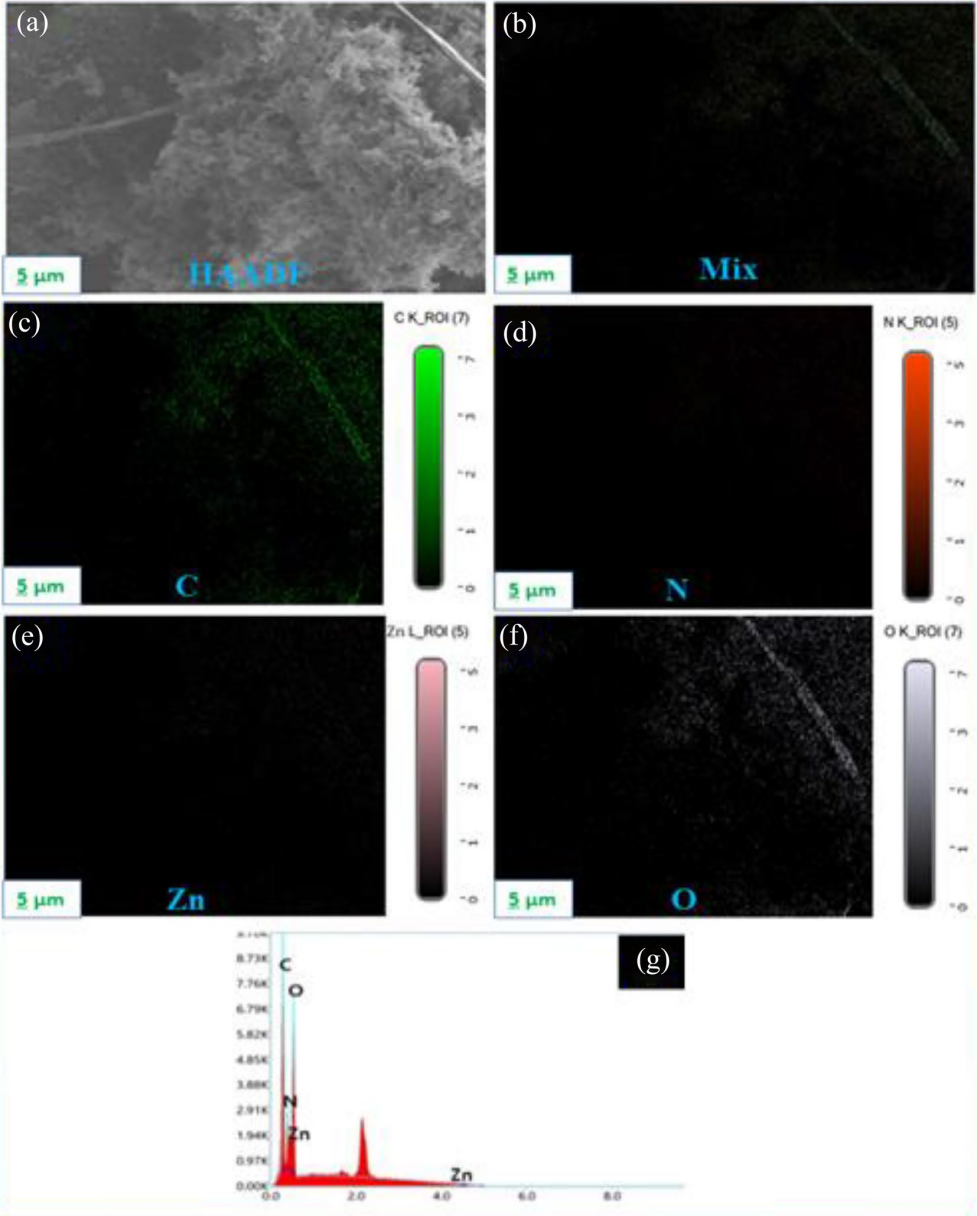
HAADF image of ZPG hybrid nanocomposite (**a**), the resulting elemental mapping investigation (**b**–**f**) of C, N, Zn, and O, and EDS analysis of ZPG nanocomposite (**g**).

Additionally, the high-angle annular dark-field picture (HAADF) (Fig. 4a) and the corresponding elemental mapping analysis (EDX) (Fig. 4b–f) display the consistent distribution of C, N, Zn, and O along the hybrid composite surface. EDS analysis (Fig. 4g) also reveals that the g-C_3_N_4_ nanosheets, PPy-NTs, and ZnO NPs exist in the hybrid composite, which agrees with XRD results (Fig. 2a).

### The anti-corrosion properties of the prepared nanocomposite coatings

**Figure 5:**
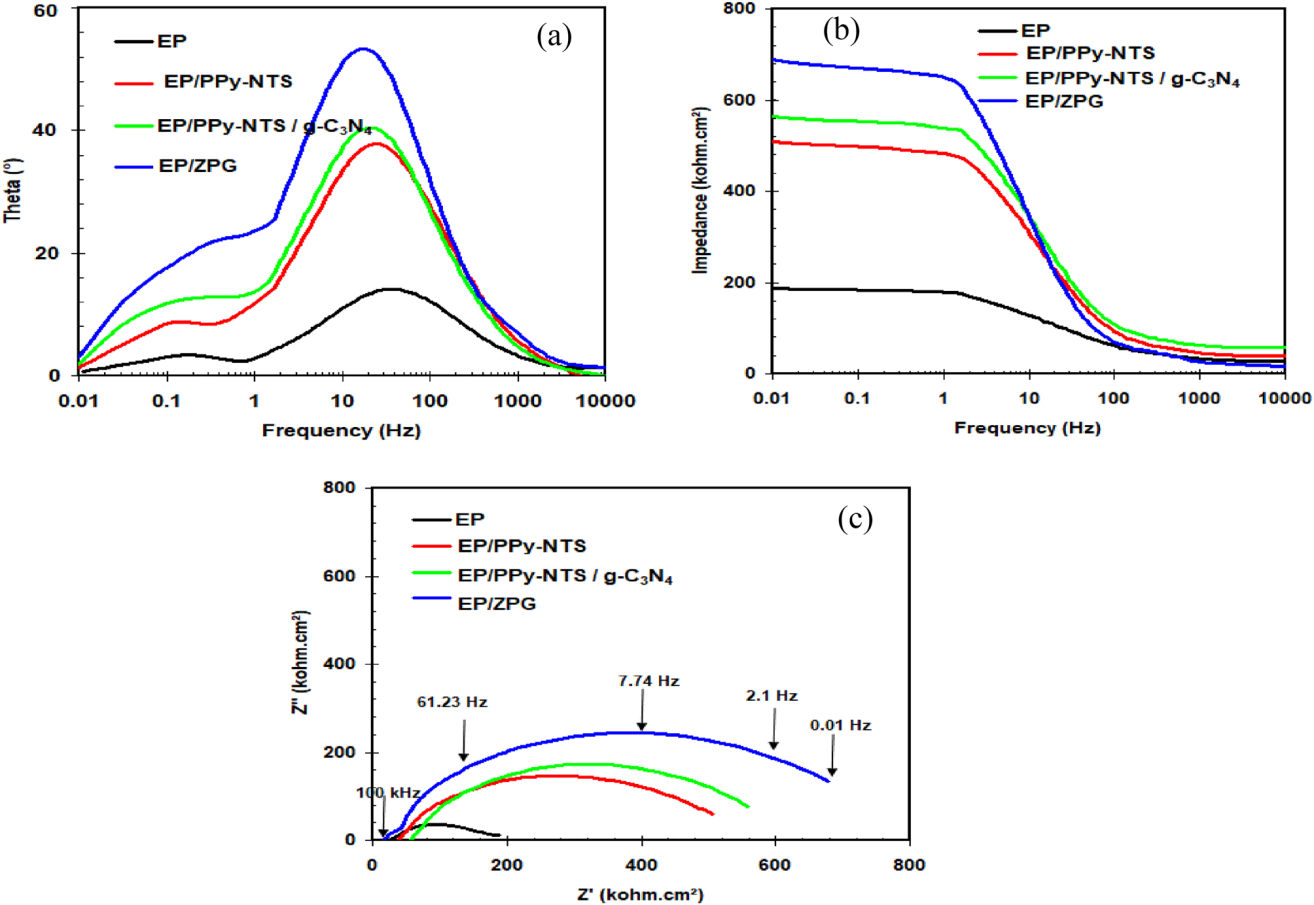
EIS spectra: (**a**) Bode-phase angle, (**b**) Bode-module, and (**c**) Nyquist plots of coated carbon steel covered with neat epoxy in the absence and presence of PPy-NTs, PPy-NTs/g-C_3_N_4_, and ZPG hybrid nanocomposite after 168 h immersion in 3.5% NaCl solutions at 298 K.

**Figure 6:**
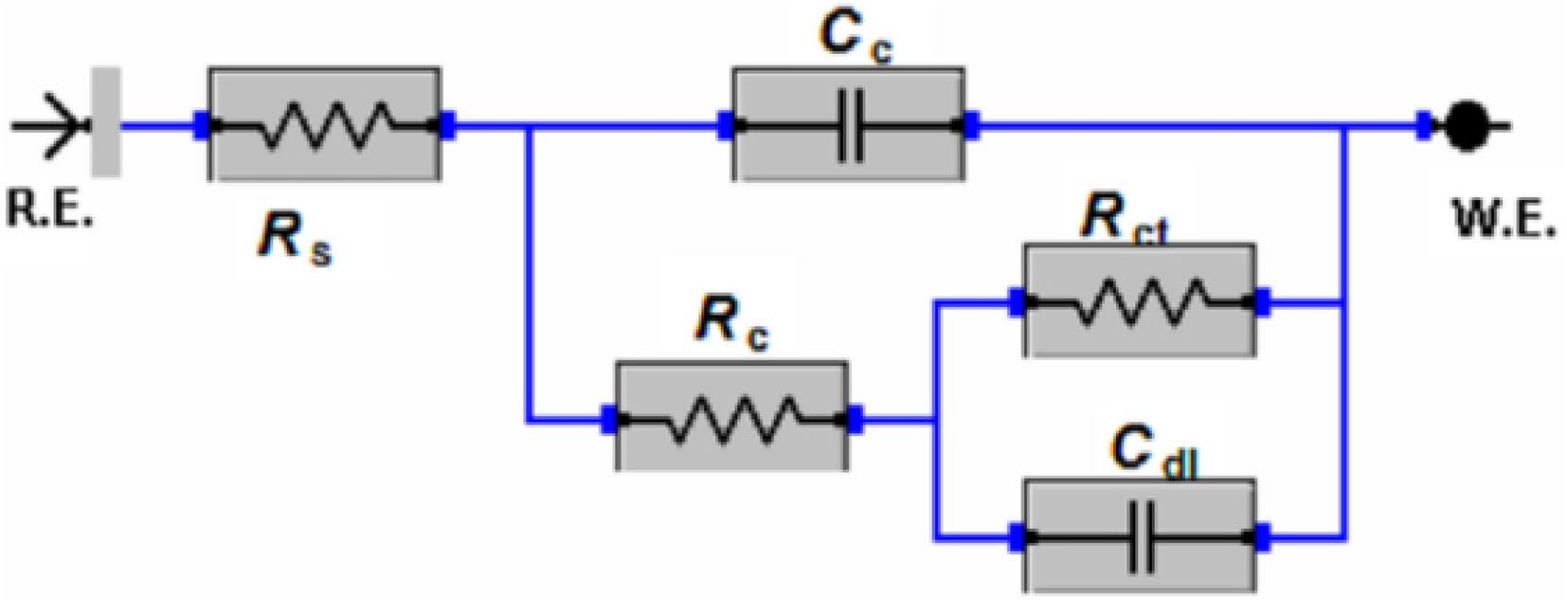
Equivalent circuit model for coated carbon steel with the neat epoxy and nanocomposite coatings.

EIS measurements were used to examine the corrosion effectiveness of nanocomposite coatings containing PPy-NTs, PPy-NTs/g-C_3_N_4_, and PPy-NTs/g-C_3_N_4_/ZnO nanocomposite. Figure 5 shows the Bode plots (Fig. 5a and b) and Nyquist plots (Fig. 5c) of epoxy resin-coated carbon steel without and with existence of the as-prepared materials after 168 h of immersion in 3.5% NaCl solutions. The case neat epoxy coating’s Nyquist plot reveals two loops^55^. The neat epoxy coating film is the subject of the first high-frequency capacitance loop^56^. The corrosion operations under the epoxy film are what cause the second loop, which occurs at low frequency^57^. Figure 6 shows the equivalent circuit that was used for illustrating the above situation. The components of this equivalent circuit, which can be found in Table 1, include coating capacitance (C_c_), the coating resistance (R_c_), charge transfer resistance (R_ct_), capacitance of the double layer (C_dl_), and solution resistance (R_s_). The values of chi-square values (χ^2^) in the Table 1 indicate goodness of fit. It is clear that the Nyquist spectrum alters when epoxy-coated carbon steel is combined with newly manufactured nano-materials. By adding as-prepared nano-materials, the sizes of both of the capacitive loops for coated carbon steel rises. This kind of system uses an equivalent circuit that is similar to the one employed for the neat epoxy coating (Fig. 6). The addition of as-prepared nanomaterials to epoxy resin coating considerably raised R_c_, R_ct_ and lowered C_dl_, C_c_ values, as shown in Table 1. The following is a list of the prepared nanocomposite coatings’ anti-corrosion effectiveness: EP/PPy-NTs/g-C_3_N_4_/ZnO ˃ EP/PPy-NTs/g-C_3_N_4_ ˃ EP/PPy-NTs. At 1.0 of PPy-NTs/g-C_3_N_4_/ZnO nanocomposite, the most effective epoxy coating effectiveness was attained. It is possible to argue that adding newly manufactured nano-materials to epoxy resin enhances the coating’s resistance to corrosion. This results from nano-particles lessening the epoxy resin's permeability, which slows the flow of corrosive ions to the surface of the metal^58^.

**Table 1:** Impedance parameters for the carbon steel coated by epoxy composite coatings immersed in 3.5% NaCl solution at 298 K.

System	χ^2^	R_c_ kΩ cm^2^	C_c_ F cm^−2^	R_ct_ kΩ cm^2^	C_dl_ F cm^−2^
**Neat EP resin**	0.00018	34.7	8.5 × 10^–8^	168.4	9.5 × 10^–8^
**EP/PPy-NTs**	0.00051	73.2	5.3 × 10^–8^	402.3	1.5 × 10^–8^
**EP/PPy-NTs/g-C**_**3**_**N**_**4**_	0.00034	94.4	1.9 × 10^–8^	512.7	1.5 × 10^–9^
**EP/PPy-NTs/g-C**_**3**_**N**_**4**_**/ZnO**	0.00038	123.6	0.5 × 10^–8^	632.5	0.4 × 10^–9^

The primary factor improving the corrosion protection of the EP/PPy-NTs/g-C_3_N_4_ coating is the mixing of PPy-NTs and g-C_3_N_4_. Where g-C_3_N_4_ nanosheets align each other parallel to the metal surface, reducing the route by which corrosive ions penetrate the surface of the metal and preventing corrosion^59,60^. On another hand, the combination of g-C_3_N_4_ and ZnO provides better corrosion protection than g-C_3_N_4_ independently. This nano-composite (EP/PPy-NTs/g-C_3_N_4_/ZnO) has different levels of protection. Morsi et al.^61^ claim that nanoparticles made of ZnO can enhance their surface area by increasing the capacity of nanocomposites to be adsorbed on metal surfaces. They can also interact with the ions released from the corrosion condition, increasing the rate chance of occurrence. Furthermore, ZnO nanoparticles may enhance steel anticorrosion properties by catalysing oxygen reduction on the exterior of steel and increasing the nanocomposite's capacity to fill certain holes and flaws on the metal surface^62^. Meanwhile, ZnO attaches corrosive species like chloride. Furthermore, g-C_3_N_4_ occupies epoxy resin pores, reducing the overall amount of corrosive ions that penetrate the metal surface^63–66^.

**Figure 7:**
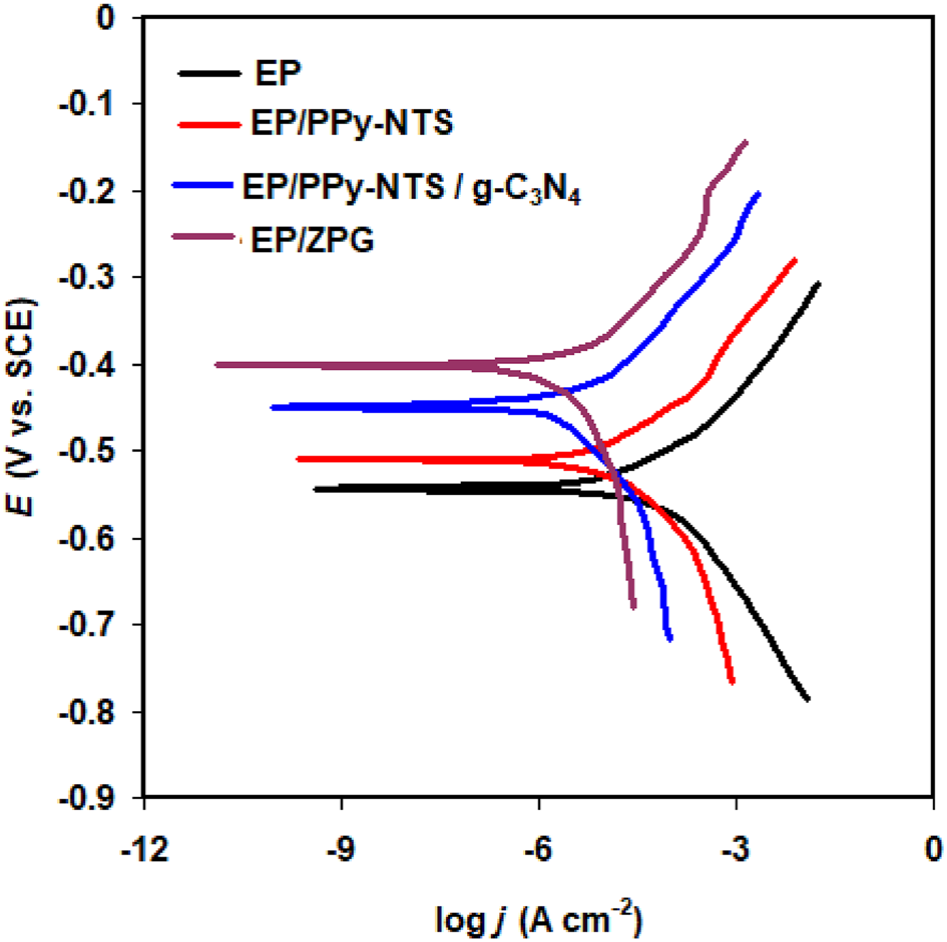
Polarization curves of coated carbon steel (dry-film thicknesses ≈ 33 µm) covered with neat epoxy in the absence and presence of PPy-NTs, PPy-NTs/g-C_3_N_4_, and ZPG hybrid nanocomposite in 3.5% NaCl solutions at 298 K.

Polarization measurements were used to assess the anti-corrosion performance of nanocomposite coatings including PPy-NTs, PPy-NTs/g-C_3_N_4_, and PPy-NTs/g-C_3_N_4_/ZnO nanocomposite (see Fig. 7). The location of the intersection of Tafel plot points^67^ was used to determine electrochemical kinetic variables (corrosion potential *E*_corr_ and corrosion current density *j*_corr_). When neat EP resin was placed in 3.5% NaCl solution, *j*_corr_ was μA cm^−2^. Carbon steel covered with PPy-NTs, PPy-NTs/g-C_3_N_4_, and PPy-NTs/g-C_3_N_4_/ZnO nanocomposites coatings, on the other hand, had significant reductions in *j*_corr_ values. *j*_corr_ was 7.3, 4.5, and 1.7 μA cm^−2^ for PPy-NTs, PPy-NTs/g-C_3_N_4_, and PPy-NTs/g-C_3_N_4_/ZnO, respectively. Furthermore, the inclusion of PPy-NTs/g-C_3_N_4_/ZnO causes a positive change in the *E*_corr_ value from − 0.543 to − 0.401 V. This result reinforces up the EIS tests, which show that adding as-prepared nanoparticles to epoxy resin coating significantly improves its anti-corrosion capabilities.

## Conclusions

Novel highly efficient anticorrosive ternary PPy-NTs/g-C_3_N_4_/ZnO nanocomposite (ZPG) nanocomposites were rationally fabricated via simple wet-impregnation technique. The performance of a novel epoxy coating (Epoxy/ZPG coating) on coated carbon steel in 3.5 wt% NaCl electrolyte was investigated. The outcomes demonstrated that ZPG in an epoxy coating exhibit superior anti-corrosion property to neat epoxy resin. This work may provide new insight into corrosion protection of epoxy coatings applications through the smart design of highly efficient materials.

## Data Availability

The datasets used and/or analysed during the current study available from the corresponding author on reasonable request.
